# ID1 marks the tumorigenesis of pancreatic ductal adenocarcinoma in mouse and human

**DOI:** 10.1038/s41598-022-17827-3

**Published:** 2022-08-08

**Authors:** Yuanxin Tang, Sheng Zhang, Jiazi Li, Chunli Wu, Qing Fan

**Affiliations:** 1 Department of General Surgery, The Fourth Affiliated Hospital of China Medical University, Shenyang, China; 2 Department of Radiation Oncology, The Fourth Affiliated Hospital of China Medical University, Shenyang, China

**Keywords:** Cancer, Cell biology

## Abstract

Pancreatic Ductal Adenocarcinoma (PDAC) is a deadly disease that has an increasing death rate but no effective treatment to now. Although biological and immunological hallmarks of PDAC have been frequently reported recently, early detection and the particularly aggressive biological features are the major challenges remaining unclear. In the current study, we retrieved multiple scRNA-seq datasets and illustrated the genetic programs of PDAC development in genetically modified mouse models. Notably, the transcription levels of Id1 were elevated specifically along with the PDAC development. Pseudotime trajectory analysis revealed that Id1 was closely correlated with the malignancy of PDAC. The gene expression patterns of human PDAC cells were determined by the comparative analysis of the scRNA-seq data on human PDAC and normal pancreas tissues. ID1 levels in human PDAC cancer cells were dramatically increased compared to normal epithelial cells. ID1 deficiency in vitro significantly blunt the invasive tumor-formation related phenotypes. IPA analysis on the differentially expressed genes suggested that EIF2 signaling was the core pathway regulating the development of PDAC. Blocking EFI2 signaling remarkably decreased the expression of ID1 and attenuated the tumor-formation related phenotypes. These observations confirmed that ID1 was regulated by EIF2 signaling and was the critical determinator of PDAC development and progression. This study suggests that ID1 is a potential malignant biomarker of PDAC in both mouse models and human and detecting and targeting ID1 may be a promising strategy to treat or even rescue PDAC.

## Introduction

Pancreatic Ductal Adenocarcinoma (PDAC), the most prevalent neoplastic disease of the pancreas, constitutes more than 90% of all pancreatic cancers^1^ and is the fourth most frequent cause of cancer-related deaths in the past few decades with poor prognosis and low survival rate^2–5^. The low survival rate of PDAC is most importantly because of the delayed clinical symptoms at advanced stages when the tumor initiates the invasion into the surrounding structures or metastasizes^3,4^. The reason of this is most due to the lack of biomarkers of PDAC carcinogenesis, which is making it more urgent to discover novel genetic or biological hallmarks to detect the development of PDAC.

scRNA-seq has been well established in recently years^6^ and has overcome the limitations of traditional microarray assays and bulk RNA sequencing methods, by sequencing the whole transcriptome at a single-cell resolution and distinguishing different cell types in tumor tissues^7^. scRNA-seq promises a better way for us to understand the tumor microenvironment and intratumor heterogeneity^8^, and enables a clearer understanding of the molecular mechanisms of tumor occurrence, and reveals the somatic mutations all throughout the course of tumor evolution^9^. PDAC is also a kind of solid tumors comprising both tumor cells and also an extremely complex microenvironment with which the tumor cells constantly interact during tumor formation^10^. Detailed characterization of the cellular composition of the tumor microenvironment is critical for the understanding of the disease and the potential therapies. To achieve that, a recent study used scRNA-seq technology to agnostically profile cell heterogeneity during different stages of PDAC progression in several well-established genetically engineered mouse models, including early and late KIC, KPfC, KPC and described the landscape of cellular heterogeneity during the progression of PDAC^11^. Another recent study complemented this cellular landscape by applied scRNA-seq on another PDAC mouse model (termed as KPC2 here)^12^. For human pancreas and related malignance study, a scRNA-seq study described the transcriptome of normal adult human pancreatic cell types and identified subpopulations of existing cell types and markers^13^. This open accessed dataset provided a perfect database as the controls of other pancreatic malignant diseases including PDAC. A more specific study targeting pancreatic preinvasive lesions analyzed tumor-associated stromal, immune, endothelial and fibroblast cells and identified signals that might support tumor development by single-cell transcriptome analysis of human PDAC^14^.

By recruiting all the above scRNA-seq databases, in the current study we integrated the normal epithelial cells, pre-malignant and malignant cancer cells from both mouse models and human PDAC tissues. We generated a completed genetic program of PDAC development and progression and identified the critical genetic genes that potentially marked the PDAC formation in mouse and human, among which ID1 is the most specific and significant one. The roles of ID in PDAC have been studied for decades by several recent studies^15–17^. The reported mechanisms included mediating cells proliferation, migration and invasion abilities, as well as the expression of HIF-1α, VEGF and MMP-9^15,16^ escaping from TGFβ tumor suppression^17^ and epithelial-mesenchymal transition (EMT)^18^. All these studies made it convincing that ID1 was significantly involved in PDAC progression. However, most of the studies above were conducted in vitro either on cell lines or by brief histology staining without any well-established validation in vivo and the detailed PDAC progression and potential upstream signaling pathways were not yet clear up to now. The association of ID1 in purified PDAC cancer cells has never been described.

In the current study, by comparative analysis, an elevated transcription of ID1 in PDAC was validated in several independent scRNA-seq and microarray datasets in both mouse and human. More importantly, the transcription levels of ID1 were dramatically increased along with the progression of PDAC. Signaling pathway and upstream regulator analysis revealed that EIF2 signaling was core signaling regulating PDAC progression and ID1 dysregulation, and this regulation genetic axis was consistent with the observations in neuroblastoma^19^. Blocking EIF2 signaling remarkably downregulated the expression level of ID1 and blunt the formation of PDAC in vitro, suggesting that ID1 was regulated by EIF2 signaling and achieved its functions in controlling tumorigenesis of PDAC.

Based on these data and in coordinate with previous studies, we assume that ID1 was a promising biomarker for the malignance of PDAC and targeting or monitoring ID1 might serve as a potential method for advanced PDAC diagnosis and therapeutic treatment in precise medicine clinically.

## Materials and methods

### Cell line

Pancreatic Carcinoma cell lines, HS-766T and PANC-1, were obtained from ATCC and were maintained in DMEM supplied with 10% FBS and the antibiotics Penicillin (100 IU) and Streptomycin (100 µg/ml).

### Gene knockdown assay

HS-766T or PANC-1 cells were split into 6-well plates and cultured until about 50% confluency. Lentivirus particles targeting specific genes (shID1 Lentiviral Particles sc-44267-V and shEIF2A Lentiviral Particles sc-35272-V, Santa Cruz Biotechnology) and the control shRNA Lentiviral particles (sc-108080, Santa Cruz Biotechnology) were pre-mixed with Polybrene (10 μg/ml) in complete medium. The cells were cultured with Lentiviral Particles (2.0 × 10^5^ IFU/well) for overnight and one well was used as a selecting control. The medium was refreshed the next day. 48 h after Lentiviral Particles infection, the cells were treated with puromycin (5 μg/ml) for 1 to 2 days until all the cells in the selecting control well were dead. For the EIF2A rescue experiments, recombinant human EIF2A protein (Recombinant Human eIF2 alpha/EIF2S1 His Protein, NOVUS Biologicals, NBP1-44467) was added into ID1 knockdown HS-766T stable line and the cells were harvested 12 h after treatment for qRT-PCR and 24 h after treatment for western blotting.

### qRT-PCR

RNA extraction was performed by RNeasy Kits (QIAGEN, 74004) from the cells after infection according to manufacturer’s instructions and RNA concentration and purify were quantified by a NanoDrop™ 2000. Reverse transcription was performed on 40 ng purified RNA by M-MLV Reverse Transcriptase system (Promega, M1701). The relative mRNA expression levels of target genes were determined by PowerUp SYBR Green Master Mix kit (Thermo Fisher Scientific, A25742). Human GAPDH was used as an internal control gene and relative expression levels were calculated by using the 2−ΔΔCt method. The sequences of specific primer pairs are listed below: ID1: forward, 5ʹ-ATTACGTGCTCTGTGGGTCTCC-3ʹ, reverse, 5ʹ-TAGTAGGTGTGCAGAGAGGAGC-3ʹ; Human eIF2A qPCR Primer Pair (SinoBiological, HP100187); GAPDH: forward, 5ʹ-ACCCAGAAGACTGTGGATGG-3ʹ, reverse, 5ʹ-TTCTAGACGGCAGGTCAGGT-3ʹ.

### Western blotting

Total proteins were extracted from the cells by RIPA Lysis and Extraction Buffer (Thermo Fisher Scientific, 89900) with Halt™ Protease and Phosphatase Inhibitor added (Thermo Fisher Scientific, 78440). Same amounts of total proteins from each sample were loaded into SDS-PAGE gels for separation and were transferred to PVDF membrane. Primary antibodies targeting specific proteins were diluted and added to the membrane and HPR conjugated secondary antibodies were used to visualize the signals. Antibodies uses are listed below: rabbit anti-Id1 polyclonal antibody (abcam, ab230679), mouse anti-eIF2A monoclonal antibody (3A7B11) (abcam, ab181467), rabbit anti-GAPDH monoclonal antibody (14C10) (Cell Signaling Technology, 2118), anti-rabbit IgG, HRP-linked antibody (Cell Signaling Technology, 7074), anti-mouse IgG, HRP-linked antibody (Cell Signaling Technology, 7076).

### Migration assay

24-well transwell cell culture chambers (Corning, 353226) were used for migration assays to determine the cell motile capacities. Briefly, 100 thousand cells in 200 μl serum-free medium were loaded into the chambers and medium with FBS as attractant was added into the bottom wells. After culturing for overnight (24 h), cells were fixed with methanol and stained with Trypan blue for visualization. Cells remaining in the transwell chambers were swiped off with cotton swabs and the cells in the bottom were regarded as migrating cells. Quantifications of the cell migration ratio were conducted by cell counting and normalized to the cell numbers of the control groups.

### Soft agar assay

The Soft Agar Assay for Colony Formation is an anchorage independent growth assay in soft agar, which is considered the most stringent assay for detecting malignant transformation of cells. Soft agar assays were performed in 24-well plate coated with 0.6% agarose diluted in DMEM. 10 thousand cells resuspended in DMEM containing 10% FBS and 0.35% agarose were plated and cultured for three weeks under standard cell culture conditions. Each group was triplicated. Cells were fixed with 10% Neutral buffered formalin and stained with 0.005% Crystal Violet for more than 1 h, and the colonies were imaged and counted by a light microscopy.

### EDU proliferation assay

Proliferation rates of cells at different condition were performed by EdU Alexa Fluor™ 488 Imaging Kit (Thermo Fisher Scientific, C10337) according to manufacturer’s instructions. In brief, a 2× working solution of EdU was prepared in complete medium and added into cell culture. After 4-h culturing, the medium was discarded, and the cells were immediately proceeded to cell fixation and permeabilization with a Fixation/Permeabilization solution (Thermo Fisher Scientific, eBioscience™ Foxp3/Transcription Factor Staining Buffer Set 00-5523-00). The reaction cocktail was prepared and added to each well and the cell plate was rocked briefly to ensure that the reaction cocktail was distributed evenly. After incubation for 30 min at room temperature protecting from light, the cocktail was washed off and the cells were analyzed by a BD Arial II cell analyzer.

### Cell apoptosis assay

Cell apoptosis of ID1 knockdown and control HS-766T cells were determined by Annexin V apoptosis detection kit (STEMCELL Technologies Scientific, 100-0338) according to manufacturer’s instructions. In brief, cell medium was aspirated, and the cells were washed with PBS for twice. The trypsinized cells were resuspended at a concentration of 1.0 × 10^6^ cells/ml in Annexin V Binding Buffer and Annexin V and 7-AAD were added at 5 μl/100 μl. The cells were incubated with Annexin V and 7-AAD at room temperature protecting from light for 15 min and washed with Binding Buffer for analysis by flow cytometry. For the flow cytometry analysis, cell populations were divided into four quadrants (Q1–Q4) with percentage quantified and populations in Q2 and Q3 were regarded as late apoptotic cells.

### scRNA-seq and microarray data accession

Open accessed scRNA-seq data and microarray data used in this study were retrieved from the NCBI Gene Expression Omnibus^20^ and the corresponding raw data from NCBI Sequence Read Archive (SRA). The accession numbers of the data on mouse PDAC mouse models, normal pancreas, early KIC, late KIC, late KPFC and late KPC, were GSE125588 and PRJNA516878. Data from another mouse PDAC model, late KPC2, were from GSE129455 and PRJNA531464. ScRNA-seq data on normal pancreas and PDAC were retrieved from GSE85241 and PRJNA337935, GSE141017 and PRJNA604712 respectively. Microarray data on human PDAC and matched control samples were from GSE16515, GSE28735, GSE32676 and GSE15471.

### Bioinformatic analysis

Bioinformatic analysis on scRNA-seq data were performed under the guidance of standard tutorial in R/R-studio. Briefly, low quality cells were removed, and the cell clusters were identified based on gene (feature) transcripts. Differential gene expression was performed to define the cell types and specific gene of each cluster and each cell type. Data integration was done when multiple datasets were needed for analysis and comparison. Ingenuity Pathway Analysis (IPA) was used to demonstrate the Canonical Signaling Pathways, Upstream Regulators and Graphic Networks of specific cell clusters based on the top differentially expressed genes. Slingshot packages were used to do the pseudotime analysis. Microarray data were also analyzed in R/R-studio after data normalization and gene ID and symbol annotation.

### Statistical analysis

All statistical data were analyzed and visualized by GraphPad Prism 8 software. Student’s two-tailed t test was used for comparing difference significance between 2 groups and a p value pf < 0.05 was considered statistically significant. Each experiment was repeated for at least three times and all results were presented as mean ± SEM.

## Results

### Access of the scRNA-seq data on PDAC mouse models

**Figure 1:**
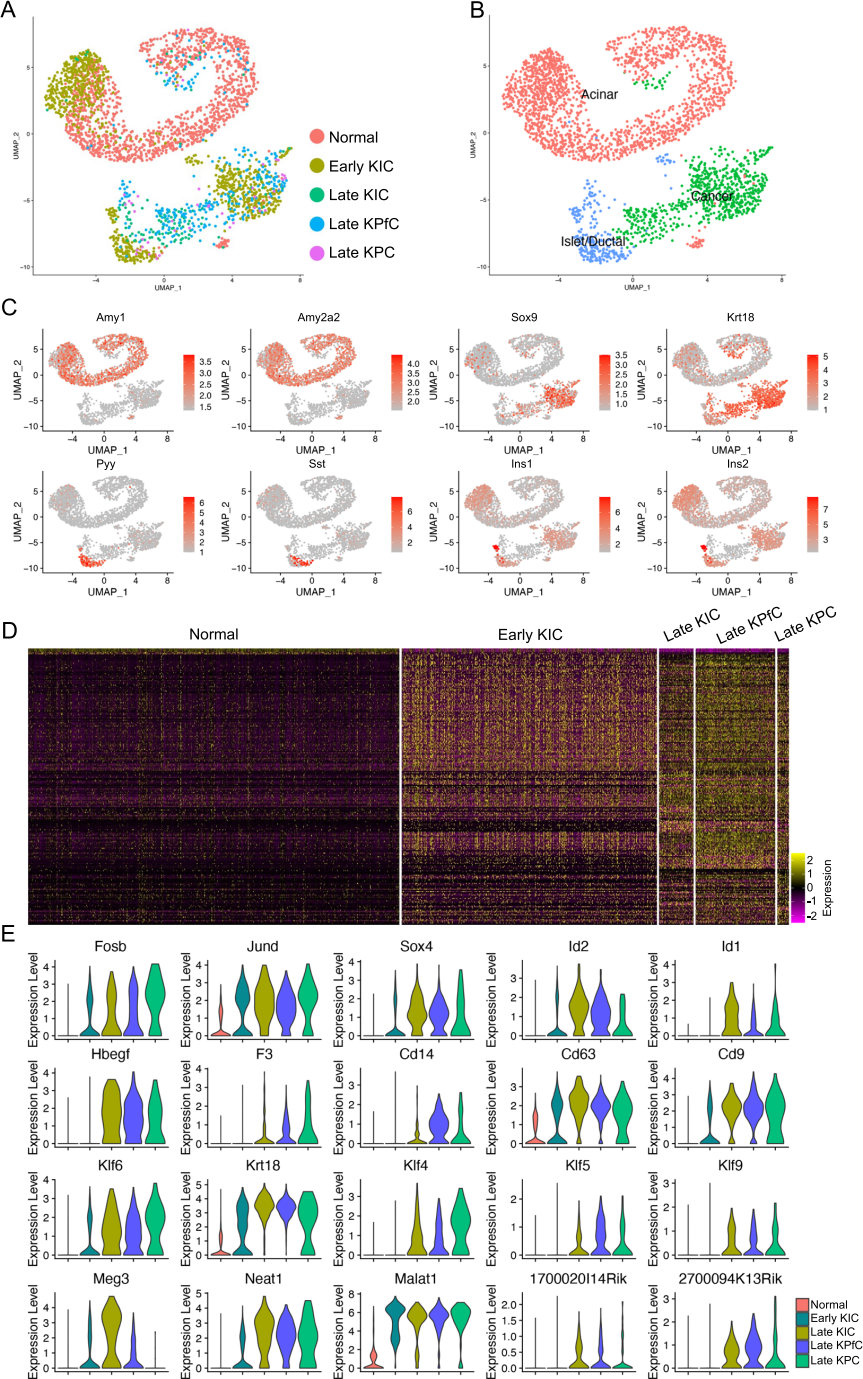
Identification of differentially expressed genes involved in the progression of PDAC development. (**A**) Cell origins of the epithelial/cancer cells from different mouse models. (**B**) Cell type identification of the integrated epithelial/cancer cells. (**C**) UMAP visualization of the expression of canonical epithelial and cancer formation related marker genes. (**D**) Heatmap of the top 100 genes of the epithelial/cancer cells of each mouse model. (**E**) Transcription levels of top five transcriptional factor, cell surface protein, kruppel like factor and LincRNA genes that were correlated with PDAC progression in mouse models.

As the development of single cell omics technologies, many of recent studies have focused more on the development and heterogeneities of malignant diseases, especially in mammary tumors. To profile cell populations and understand the phenotypic changes during PDAC progression, a recent study used scRNA-seq to agnostically profile cell heterogeneity during different stages of PDAC progression in genetically engineered mouse models^11^, including normal and four PDAC models, early KIC, late KIC^21^, late KPfC^22^ and late KPC^23^. Violin plots were used to visualize the cells qualities and the low-quality cells or doublets were removed (Supplementary Fig. S1A,B). Batch effects were corrected and retained cells were clustered and visualized by UMAP (Supplementary Fig. S2A,B). Cell types were identified by checking canonical cell type specific markers (Supplementary Fig. S2C,D)^14^. Batch correction was confirmed by split UMAPs (Supplementary Fig. S3A), and cell-type identification was confirmed by the expression of the cell type markers visualized by violin plots (Supplementary Fig. S3B). Gene expression patterns were specificized by heatmap of top 100 genes of each defined cell type (Supplementary Fig. S2E). We then subset and clustered the epithelial lineage cells and identified the normal acinar (Amy1+ and Amy2a2+) and islet/ductal cells (Pyy+ and Sst+, Ins1+ and Ins2+) as well as a cancer cell cluster with normal epithelial lineage marker expression excluded (Sox9+ and Krt18+) (Fig. 1A–C). All the cells in cancer cluster were contributed by the PDAC cancer models (Fig. 1A). We also performed the differential expression (DE) analysis on the epithelial cell lineages among the different models and found the DE genes between normal and PDAC models (Fig. 1D). To demonstrate the genetic programs associated with the development of PDAC, we identified the genes dysregulated in the PDAC models and visualized the top genes by violin plots (Fig. 1E). Several transcriptional factor genes, including Fosb^24^, Jund^25^ and Sox4^26,27^ were reported to be associated with development, survival or outcome of pancreatic cancer in human. Among these top transcriptional factor genes, the most correlated gene was Id1, expression of which increased along with development of PDAC with rare transcription in normal and less malignant epithelial cells (Fig. 1E). Besides these transcriptional factor genes, multiple cell surface genes, Keratin and Kruppel Like Factor genes as well as several long intervening noncoding RNAs (LincRNA) were also upregulated in the PDAC models (Fig. 1E). These genes were also commonly reported to be correlated with tumor development^28^, grading^29^, suppression^30,31^, prognosis^32^, tumorigenicity^33^ and risk^34^ of pancreatic cancer. LincRNA Malat1 promotes aggressive pancreatic cancer proliferation and metastasis via multiple reported signaling pathways^35–37^. Another LincRNA, Meg3, was commonly reported to be a tumor suppresser gene^38^ and was also dramatically associated with PDAC development^39^ and malignance^40,41^.

**Figure 2:**
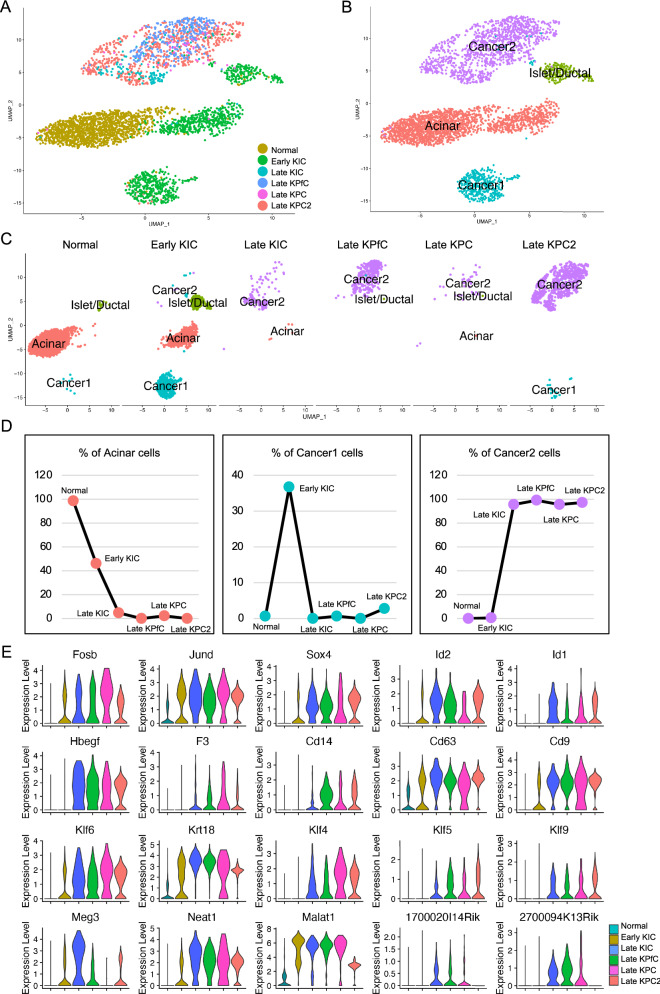
Validation of differentially expressed genes involved in the progression of PDAC development in another model. (**A,B**) Integration of the epithelial/cancer cells from six mouse models (**A**) and cell type definition of the integrated epithelial/cancer cells (**B**). (**C**) Cell distribution of each cell type in each mouse model. (**D**) Cell percentage of normal epithelial cell and cancer cell clusters in each mouse model. (**E**) RNA levels of the top genes corelated with PDAC progression in the six mouse models.

By the similar way, we retrieved one more scRNA-seq dataset on another mouse model of late KPC (late KPC2)^12^. Cells were clustered (Supplementary Fig. S4C) after quality control (Supplementary Fig. S4A,B) and cell types were defined based on the cell type specific maker gene expression (Supplementary Fig. S4D–F). The epithelial cell lineages (Epi_lin) were extracted and integrated with the Epi_lin extracted above (Fig. 2A). Based on the gene transcriptional patterns of the integrated data (Supplementary Fig. S5A,B), two normal epithelial cell clusters, acinar cell cluster and islet/ductal cell cluster, and two malignant cell clusters were identified (Fig. 2B). The normal epithelial cells were mostly contributed by the normal pancreases tissue and early KIC (Fig. 2C). To confirm that, we quantified the cell percentage of each cell cluster in each mouse model (Fig. 2D). Acinar cell percentage was decreased while percentage of cancer cell Cluster 2 was dramatically increased with the progression of PDAC. Cancer cell Cluster 1 showed highest percentage in early KIC only, suggesting that cluster 1 cancer cells potentially marked the initiation of PDAC in these mouse models. We also validated the transcription of these dysregulated transcriptional factor genes, cell surface protein genes, Kruppel Like Factor genes and LincRNA genes in these PDAC mouse models (Fig. 2E) and many other top genes (Supplementary Fig. S6). These genes showed increased expression in the PDAC models, and the most notable gene was Id1, which showed visible transcription only in late malignant PDAC models by violin plots (Fig. 2E).

### Id1 marks the development of PDAC in mouse models

**Figure 3:**
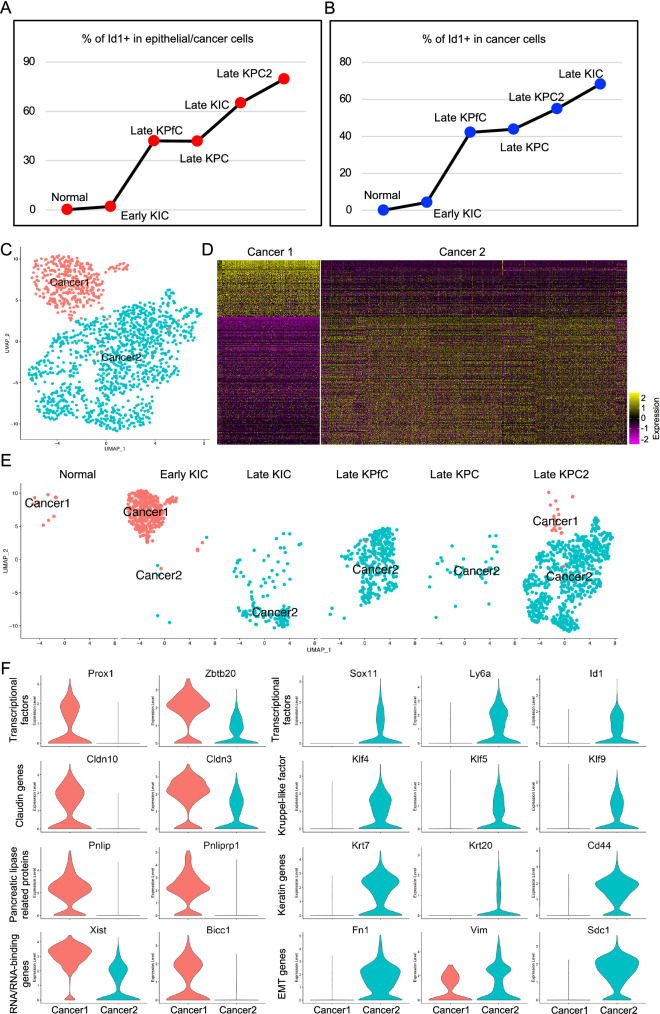
Cancer cell subtypes and corresponding marker genes involved in the progression of mouse PDAC. (**A,B**) Percentage of Id1+ cells in total epithelial/cancer cells (**A**) and cancer cells clusters in each mouse model (**B**). (**C**) Extraction and re-clustering of the cancer cells. (**D**) Heatmap of top 500 genes of each cancer cell subpopulation. (**E**) Cell distribution of each cancer cell subpopulation in each mouse model. (**F**) Violin plot visualization of the representative top genes of each cancer cell subpopulation.

Significant increase in Id1 transcription in mouse models implied that Id1 might be involved in the tumorigenesis of PDAC. To further confirm that, we quantified the percentage of the epithelial/cancer cells with Id1 transcription at Id1 > 1 and found a gradual increased percentage along with the progression of PDAC (Fig. 3A).

To demonstrate the profile differences of two cancer cell clusters identified above, these cells were subset (Fig. 3C), and surprisingly, Id1 transcription in these cancer cells was also significantly associated with the progression of PDAC (Fig. 3B). Again, cancer cells in Cluster 1 were confirmed to be contributed by normal pancreases and early KIC, while Cluster 2 by the other more malignant models (Fig. 3E). Distinct DE patterns of two cancer cell clusters were visualized by heatmap on top 500 genes (Fig. 3D). These patterns were visualized by violin plots of top genes of each cancer cluster, including transcriptional factor genes, Claudin genes, Pancreatic lipase related genes and RNA/RNA-binding genes in Cluster 1, as well as including transcriptional factor genes, Kruppel Like Factor genes, Keratin genes and Epithelial-mesenchymal transition (EMT) related genes in Cluster 2 (Fig. 3F). Notably, many of the specific genes in Cluster 1 were associated with normal development and functions of pancreas epithelial cells^42–47^. In Cluster 2 the EMT related genes, including Fn1, Vim and Sdc1, were significantly upregulated, suggesting that Cluster 2 underwent active EMT, one of the major features of cancer progression. The transcriptional genes, Kruppel Like Factor genes and Keratin genes, as mentioned above, were closely correlated with tumor development, prognosis and grading of pancreatic cancer (Fig. 3F). More interestingly, Id1 gene was also found to be remarkably upregulated in Cluster 2, suggesting that Id1 was a consistent marker for PDAC progression and malignant cancer cells in mouse models.

### Id1 marks differentiation potentials of cancer cell clusters

**Figure 4:**
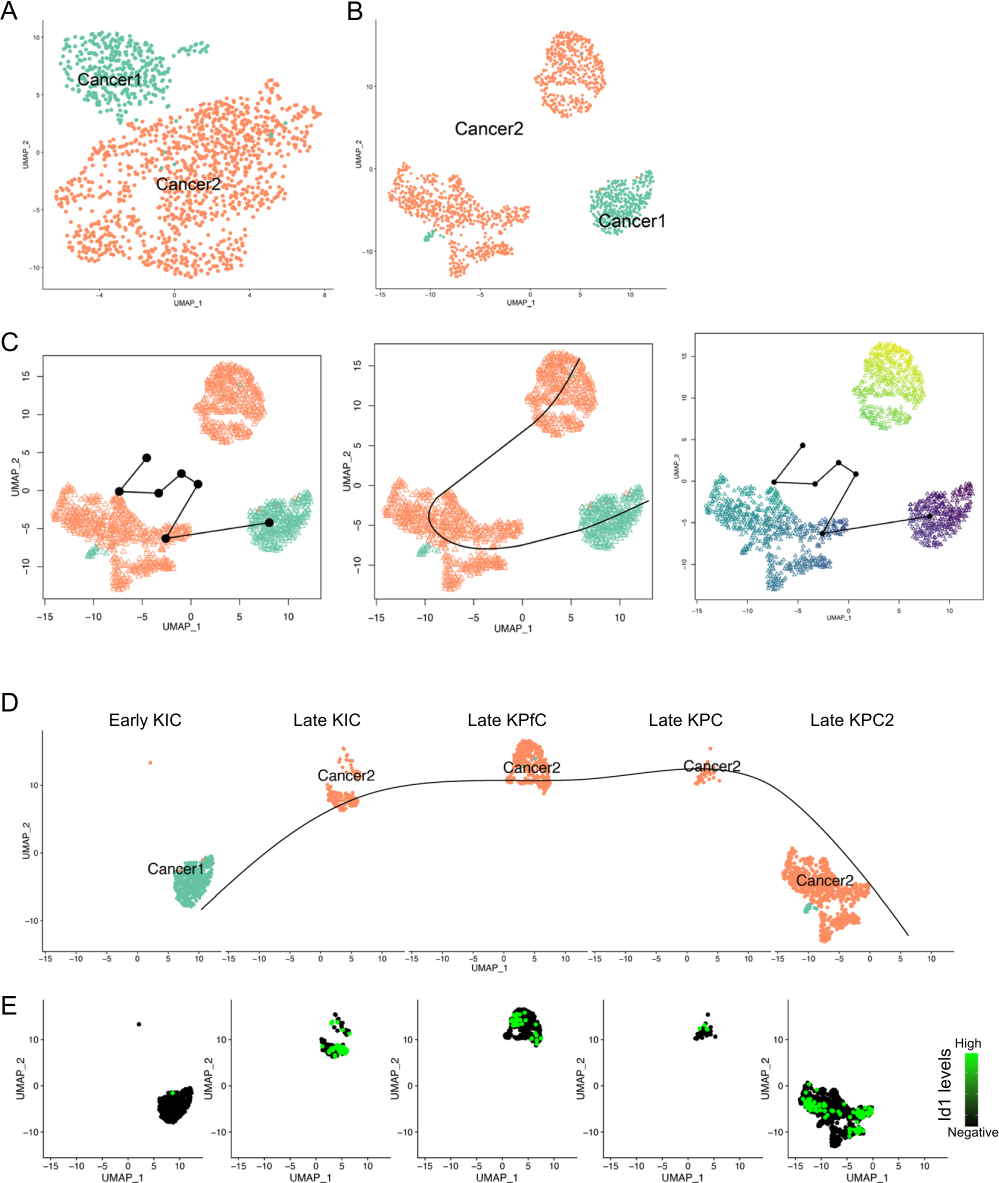
Diversity and differential potentials of the cancer cell subpopulations. (**A,B**) Cancer cell subcluster distribution (**A**) and reclustering in Slingshot (**B**). (**C**) Trajectory analysis of cancer cells revealed the differentiation direction of the cancer cell subclusters. (**D**) Distribution of cancer cells from each mouse model in the principal curves. (**E**) The increase of Id1 transcription was consistent with the differentiation direction of the cancer cell subclusters.

As two cancer cell clusters were identified, and Cluster 1 showed more normal epithelial cell features, while Cluster 2 demonstrated more malignant features, we assumed that Cluster 1 was derived from normal epithelial cells and gave rise to Cluster 2. To confirm that, we applied Slingshot packages^40^ to determine the differentiation potentials of the cancer cell clusters. The cancer cells were loaded and visualized in Slingshot (Fig. 4A) and re-clustered, and Cluster 2 were divided into two major subclusters (Fig. 4B). The trajectory analysis revealed that the differentiation was started from Cluster 1 to one subcluster of Cluster 2 and finalized at the other subcluster of Cluster 2 (Fig. 4C). Principal curve in split UMAP also confirmed that the differentiation was initiated from Cluster 1 and finalized at Cluster 2 (Fig. 4D). Surprisingly, Id1 transcription levels were increased with the principal curve of the differentiation potential (Fig. 4E), further suggesting that Id1 was critical for the progression of the cancer. The transcription of the other signature genes of Cluster 1 and Cluster 2 was also illustrated by UMAPs (Supplementary Fig. S7A,B).

**Figure 5:**
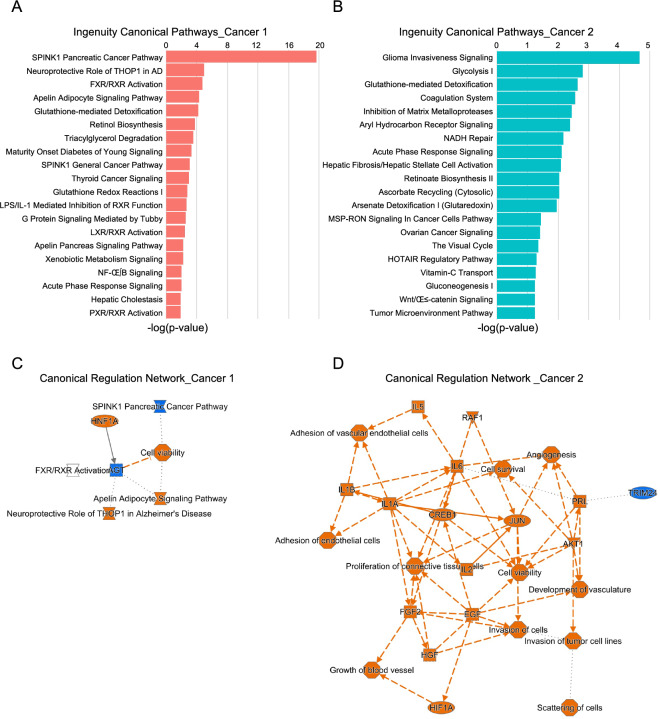
Ingenuity canonical pathways and related pathway networks of the cancer subclusters. (**A,B**) Top 20 ingenuity canonical pathways of cancer cell subcluster 1 (**A**) and cancer cell subcluster 2 (**B**) determined by IPA analysis. (**C,D**) Canonical regulation networks of cancer cell subcluster 1 (**C**) and cancer cell subcluster 2 (**D**) based on ingenuity canonical pathways and upstream regulators by IPA analysis.

To evaluate the different regulation potentials of the two cancer cell clusters, we input the upregulated genes of both Cluster 1 and Cluster 2 into Ingenuity Pathway Analysis (IPA). The top canonical pathways of Cluster 1 included pancreatic cancer initiation pathways, including SPINK1 Pancreatic Cancer Pathway^48^, and multiple normal pancreatic function related pathways (Fig. 5A,C), including FXR/RXR Activation^49,50^, Apelin Adipocyte Signaling Pathway^51,52^ and Retinol Biosynthesis^53,54^. Multiple malignant cancer related canonical pathways and upstream regulators were with Cluster 2, including Glioma Invasiveness Signaling, Ovarian Cancer Signaling, Tumor Microenvironment Pathway Invasion of tumor cell lines and Cell survival signaling pathways (Fig. 5B,D). The top upstream regulators of these two clusters were also listed (Supplementary Fig. S7C,D).

### Upregulation of ID1 in human PDAC cancer cells

As Id1 gene was significantly upregulated and marked the progression of PDAC in mouse models, to validate this in human, we first accessed a recently published scRNA-seq dataset on normal human pancreas^13^. After quality control (Supplementary Fig. S8A,B), we clustered the cells and defined the major cell types based on canonical cell type marker gene expression (Supplementary Fig. S8C–E). The epithelial cells were the subset and clustered for further analysis (Supplementary Fig. S8F). Similarly, another published scRNA-seq dataset on primary human PDAC was retrieved and quality controlled (Supplementary Fig. S9A,B). Cancer cells were extracted and clustered (Supplementary Fig. S9F) after cell type definition (Supplementary Fig. S9C–E).

**Figure 6:**
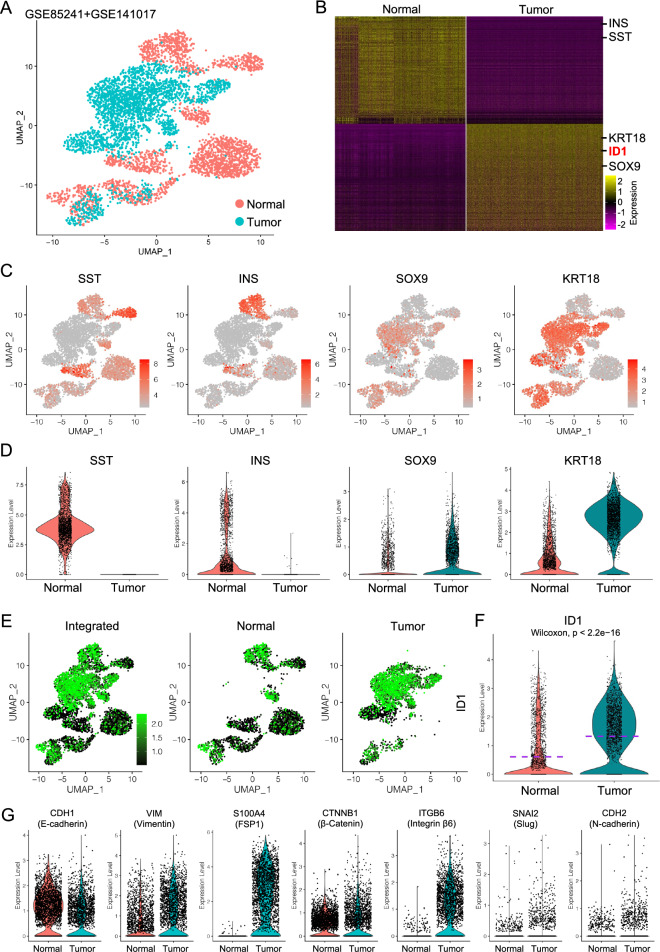
Upregulation of ID1 in human PDAC tumor cells. (**A**) Integration of the normal human pancreatic epithelial and PDAC cancer cells. (B) Heatmap of top 500 genes of normal human pancreatic epithelial cells and PDAC cancer cells. (**C,D**) UMAP (**C**) and violin plot (**D**) visualization of the expression of normal pancreatic epithelial marker genes and cancer related epithelial marker genes. (**E**) UMAP visualization of ID1 expression in integrated data and spilt normal epithelial cells and PDAC cancer cells. (**F**) Violin plot comparison of ID1 transcription in normal epithelial cells and PDAC cancer cells. (**G**) Violin plot visualization of EMT related gene expression in normal epithelial cells and PDAC cancer cells.

Extracted normal epithelial cells and cancer cells were then integrated (Fig. 6A), and DE analysis were performed and visualized by heatmap of top 500 genes to determine the gene expression patterns of each cell condition (Fig. 6B). Notably, the expression levels of normal pancreatic epithelial cell markers, SST and INS, were specifically higher compared to cancer cells, suggesting the normal functions of these cells, while malignant marker genes, SOX9 and KRT18, were remarkably upregulated in cancer cells (Fig. 6B–D). More surprisingly, ID1 was the top one transcriptional factor gene (Fig. 6B) and was dramatically upregulated and specific in cancer cells (Fig. 6E,F), suggesting that ID1 might be critical for the PDAC development in human. Also, the activated EMT features were also identified in the PDAC cancer cells, as shown by reduced epithelial marker genes expression and upregulated mesenchymal and EMT marker gene expression (Fig. 6G), suggesting a potential role of ID1 in EMT of PDAC cancer cells in human.

**Figure 7:**
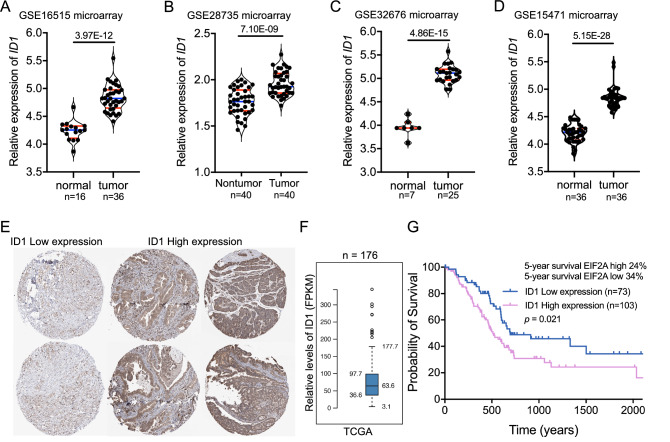
ID1 was upregulated in PDAC tumor tissues and was associated with poor survival of PDAC. (**A**–**D**) Comparison of ID1 expression in multiple microarray data of PDAC tumor tissues and matched non-tumor tissues. (**E**) Differential expression of ID1 in different PDAC patients. Relative expression of ID1 (FPKM) (**F**) and survival probability of patients with different ID1 expression (**G**) in TCGA dataset.

To further confirm the elevated levels of ID1 in PDAC, we accessed multiple microarray datasets based on human PDAC and matched normal tissues. Consistent to the data in scRNA-seq data, in all the microarray datasets ID1 was significantly upregulated in PDAC tumors compared to matched nontumor tissues (Fig. 7A–D). In the TCGA (The Cancer Genome Atlas Program) dataset, tumor tissues from different PDAC patients showed different ID1 expression levels (Fig. 7E,F), and patients with high ID1 expression had significant lower overall survival years compared to those with lower ID1 expression (Fig. 7G). Based on these data, we assumed that ID1 was the key transcriptional factor gene which was closely involved in the tumorigenesis od PDAC in human.

### ID1 deficiency reduced PDAC tumor progression in vitro

**Figure 8:**
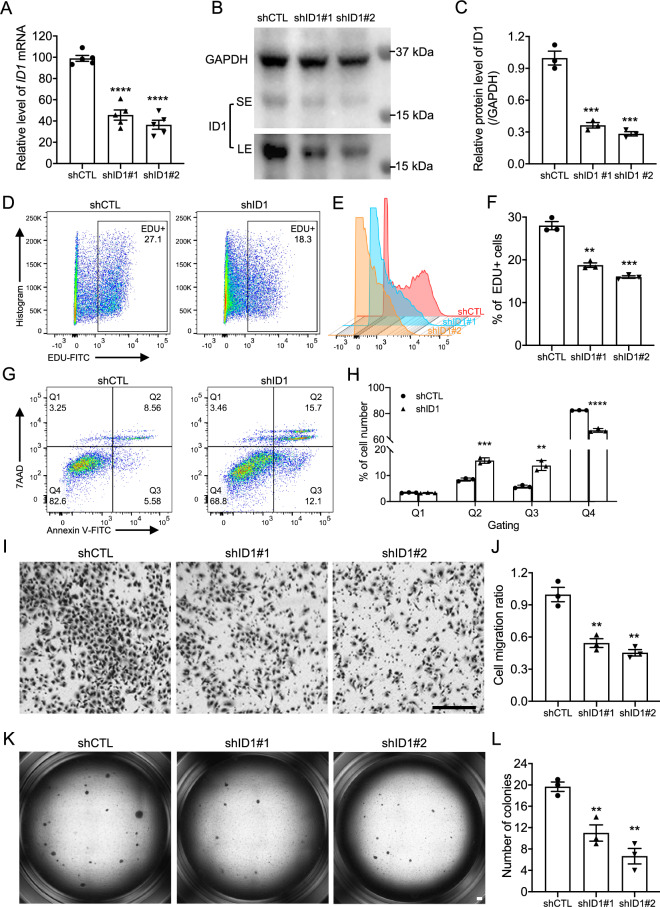
ID1 deficiency attenuated tumor formation related phenotypes. (**A**) qRT-PCR of the mRNA levels of *ID1* gene in HS-766T cells after knockdown assays (n = 5). (**B,C**) Western blotting (**B**) and matched quantification (**C**) confirmed the efficiency of the knockdown assays (n = 3). (**D,E**) Flow cytometry analysis of EDU labeled cell proliferation assay in HS-766T cells after knockdown assays. (**F**) Percentage quantification of the EDU+ proliferating cells after *ID1* knockdown in HS-766T cells (n = 3). (**G,H**) Cell apoptosis of HS-766T cells after *ID1* knockdown (**G**) and cell percentage of each gated cell population ((**H**), n = 3). (**I,J**) Cell migration (**I**) and the migration ratio (**J**) of HS-766T cells after *ID1* knockdown (n = 3). (**K,L**) Soft agar assays (**K**) and colony number quantification (**L**) of HS-766T cells after *ID1* knockdown (n = 3). Three independent repeats or more were performed for each experiment. *SE* short exposure, *LE* long exposure. Scale bar, 50 μm. **p < 0.01; ***p < 0.001, ****p < 0.0001.

To confirm the functions of ID1 in PDAC progression, *ID1* knockdown assay was conducted on HS-766T cells, a commonly used human pancreatic carcinoma cell line. qRT-PCR and western blotting confirmed the knockdown efficiency at RNA levels (Fig. 8A) and protein levels (Fig. 8B,C). The flow cytometry-based EDU labeling assay was used to determine the cell proliferation rate of the HS-766T cells^55^. *ID1* knockdown significantly decreased HS-766T cell proliferation (Fig. 8D,E) and quantified percentage of the EDU+ cells further confirmed that (Fig. 8F). *ID1* knockdown also increased HS-766T cell apoptosis determined by Annexin V assay (Fig. 8G,H). In vitro cell migration is a commonly used assay to evaluate cancer cell motility and malignance^56^. We found that *ID1* knockdown caused significant decrease in HS-766T cell migration (Fig. 8I,J). The soft agar colony formation assay is a well-established method for characterizing the carcinogenesis capability in vitro and is one of the most stringent tests for malignant transformation in cells^57^. ID1 deficiency significantly reduced the colony formation capacities of HS-766T cells (Fig. 8K,L), suggesting that ID1 was required for the carcinogenesis of the normal pancreatic epithelial cell. The functions of ID1 in PDAC progression were also verified in another PDAC cell line, PANC-1, by cell proliferation, migration and colony formation assays (Supplementary Fig. S10A–H).

### EIF2 signaling was the core pathway to regulate ID1 in of PDAC

**Figure 9:**
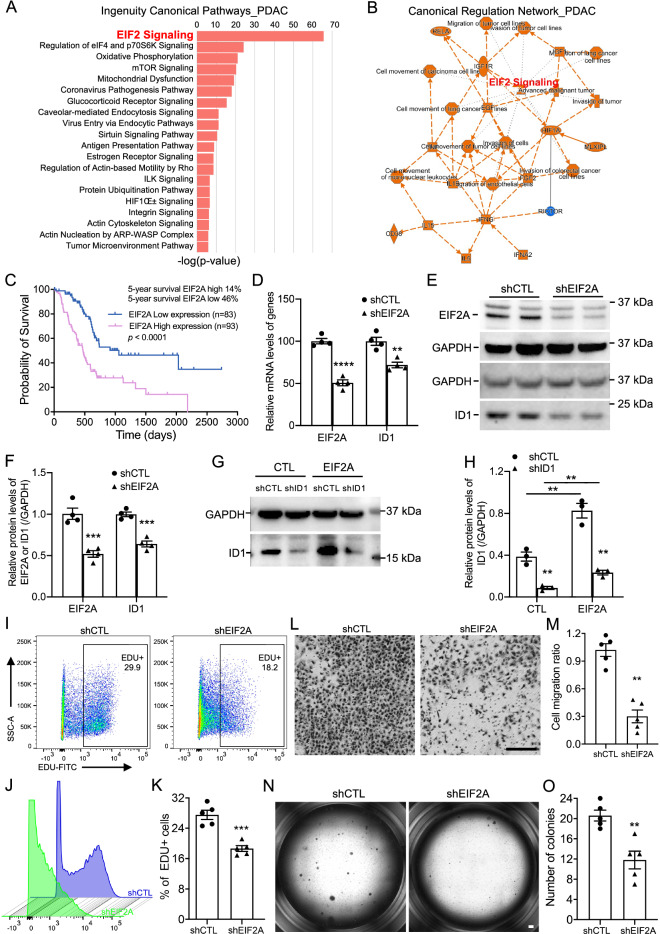
Blocking EIF2A signaling blunt tumorigenesis in vitro. (**A,B**) Top 20 ingenuity canonical pathways (**A**) and canonical regulation network (**B**) on the DE genes of PDAC cancer cells. (**C**) Survival probability of PDAC patients with different EIF2A expression in TCGA dataset. (**D**) Relative mRNA levels of *EIF2A* and *ID1* genes in HS-766T cells after *EIF2A* knockdown by qRT-PCR (n = 4). (**E,F**) Representative western blotting (**E**) and relative protein levels (**F**) of EIF2A and ID1 after *EIF2A* knockdown (n = 4). (**G,H**) Representative western blotting (**G**) and relative protein levels (**H**) of ID1 after *ID1* knockdown and recombinant EIF2A protein treatment (n = 3). (**I,J**) Cell proliferation of HS-766T cells after *EIF2A* knockdown by flow cytometry analysis on EDU labeled cells. (**K**) Quantification of the EDU+ proliferating cells after *EIF2A* knockdown in HS-766T cells (n = 5). (**L,M**) Cell migration (**L**) and the migration ratio (**M**) of HS-766T cells after *EIF2A* knockdown (n = 5). (**N,O**) Representative images of soft agar assays (**N**) and colony number quantification (**O**) of HS-766T cells after *EIF2A* knockdown (n = 5). Three independent repeats or more were performed for each experiment. Scale bar, 50 μm. **p < 0.01; ***p < 0.001, ****p < 0.0001.

To determine the upstream regulators and related canonical signaling pathway involved in the ID1 dysregulation and tumor formation, we applied IPA analysis on the DE genes of PDAC cancer cells from the scRNA-seq analysis. Many top canonical pathways and upstream regulators were closely associated with pancreatic cancer (Fig. 9A,B) and the core and the most significant ingenuity canonical pathway was EIF2 signaling (Fig. 9A,B).

As EIF2A is the major component of EIF2 signaling, we verified the expression of EIF2A gene in TCGA dataset and found significant association of EIF2A expression levels with poor survival of PDAC patients (Fig. 9C). These data suggested a core role of EIF2A in determining the malignancy of PDAC. To confirm that, we performed *EIF2A* knockdown assay on HS-766T cells. *EIF2A* knockdown led to a significant decrease in mRNA and protein levels of EIF2A and ID1 (Fig. 9D–F). In addition, adding EIF2A protein to the HS-766T cells significantly induced the elevation of ID1 in HS-766T cells, further validating that ID1 gene was regulated by EIF2 signaling in PDAC cancer cells (Fig. 9G,H). As expected, *EIF2A* knockdown remarkably reduced HS-766T cell proliferation confirmed by flow cytometry-based EDU labeling assay (Fig. 9I–K) and decreased cell motility capacity determined by migration assay (Fig. 9L,M). Also, the colony formation efficiency of HS-766T cells was significantly decreased after *EIF2A* knockdown (Fig. 9N,O), suggesting a decreased ability of transformed cells to grow independently of a solid surface and reduced hallmark of carcinogenesis of these cells. All these data suggested a core regulation potential of EIF2A in tumor formation gene expression patterns and tumorigenesis of PDAC.

## Discussion

Pancreatic ductal adenocarcinoma (PDAC), a pancreatic cancer accounts for more than 90% of all pancreatic malignances, is a devastating malignancy with extremely poor diagnosis and prognosis, as shown by 5-year survival rate of around 5–7% and 1-year survival rate of less than 20% of total cases of all stages^58–60^. The incidence of PDAC is expected to rise further in the future, and the death rates continued to increase^5^. The low survival rates of PDAC patients are principal since the fact arises that PDAC tumors progress rapidly with very few specific symptoms and are thus usually diagnosed at advanced stages for most patients, as well as aggressive nature of the tumor, and the resistance to chemotherapy and radiotherapy^61,62^. As a result, there is an urgent need to develop accurate markers of pre-invasive pancreatic neoplasms so as to facilitate prediction of cancer risk and to help diagnose the disease at an earlier stage^63^. However, although many studies and clinical trials have sought to identify inexpensive, noninvasive, or minimally invasive biomarkers with high sensitivity and specificity for PDAC to improve early diagnosis and subsequent treatment^64^, screening for early diagnosis of PDAC remains challenging and identifying a highly accurate, low-cost screening patient-acceptable test for early PDAC for use in clinical practice remains an important unmet need^58,63^.

In the current study, by combining multiple datasets, we comprehensively identified that ID1 is a promising pre-malignant biomarker for the screening and monitoring of PDAC development progression. ID1 is a helix-loop-helix (HLH) protein that can form heterodimers with members of the basic HLH family of transcription factors^65^, the overexpression of which has been reported in many types of cancers in either transcriptional or translational levels, including breast, prostate and pancreatic cancers^66–68^. Several studies have also reported that ID1 has potential to be a prognosis factor associated with patients’ survival for PDAC^16,69^. A histology study from several years ago revealed that ID1 overexpression was significantly related with tumor angiogenesis, higher density of intratumoral vessel, but not associated with a poorer prognosis or a higher proliferative potential in human pancreatic cancers^69^. However ten years later, another study determined that overexpression of ID1 was found to be closely correlated with that of VEGF, an endothelial growth factor that has a central role in cancer angiogenesis^70,71^, and to be associated with high microvessel density and patient survival in PDAC^16^. These studies shared most in common but had a few inconsistent findings. A most recent study has applied 2-Methoxyestradiol (2-ME), a natural derivative of estradiol with ability of suppressing ID1 levels in cell culture, in the function study of ID1 in pancreatic cancer development and progression in vitro^15^. Elevated cell proliferation, migration, invasion and tumor xenograft growth caused by ID1 overexpression were blunt by 2-ME treatment in pancreatic cancer cell line^15^. TGFβ signaling mediates tumor-suppressive or tumor-progressive effects^72^ and causes apoptosis in premalignant cells^73,74^. Cancer cells derived from PDAC tumor tissues that displayed an active TGFβ pathway averted cell apoptosis by transcriptional dysregulation of ID1, also known as an inhibitor of progenitor cell differentiation^75^. Transcriptional induction of ID1 uncoupled TGFβ-induced Epithelial-mesenchymal transition (EMT) from apoptosis. The dysregulation of ID1 expression resulted from a diverse set of alterations, including PI3K–AKT signaling pathway mutations^17^. These findings shed light on the crucial roles of ID1 in the development of pancreatic cancers and the data were impressive and informative, however many of them were either just bulk histological views on the autopsy slides or plastic phenotypes of cell lines without proper in vivo validation. In this current study, by retrieving multiple scRNA-seq datasets on both mouse models and human PDAC tissues, we purified the epithelial cell lineages and cancer cells and revealed the gene programs of cancer cells at different stages of PDAC progression. Consistently in both mouse and human, ID1 showed a dramatically increased transcription with the progression of PDAC, which was further confirmed by multiple microarray datasets on PDAC and matched non-tumor tissues.

Besides these pancreatic cancer specific findings, ID1 was also found to showed critical roles in Epithelial–Mesenchymal transition (EMT)^76,77^, one of the critical features of the tumorigenic process^78^. As identified in other cancers, ID1 transcription levels in the cancer cells of both mouse models and human PDAC tissues were closely correlated with cancer cell EMT procession, as well as other tumor-formation related cell phenotypes, including cell proliferation, migration, and colony formation in vitro. Signaling pathway analysis revealed that the functions of ID1 dysregulation in tumor progression were mainly mediated by EIF2 signaling, which was commonly reported as a promising target in cancer therapy^79^. EIF2 signaling activities were reported to be elevated in PDAC tumors, as shown by higher phosphorylated EIF2A, p-EIF2A, expression in PDAC tissues compared to normal pancreas tissues. High PERK and p-eIF2α expression was correlated with shorter overall survival and high p-EIF2A levels and lymph node metastasis were independent prognosticators for survival of PDAC patients^80^. The activation of the EIF2 signaling pathway, shown by the increased levels of key components of the EIF2 signaling pathway, p-PERK, p-EIF2A, and ATF4, significantly correlated with the knockdown of PUM1, the expression levels of which were higher in PDAC tissues than in adjacent tissues and were significantly associated with Tumor Node Metastasis (TNM) stage and overall survival time of PDAC patients^81^. One recent histological investigation revealed a significant down-regulation of four specific EIF subunits, namely EIF1, EIF2D, EIF3C and EIF6, suggesting not only EIF2 signaling, but also other members of the EIF family were of relevance in pancreatic tumor biology and might play a major role in translational control in PDAC^82^. In our study, EIF2 signaling pathway was the core upstream regulator of ID1 expression and PDAC development and progression. Blocking EIF2 signaling significantly reduced the expression levels of ID and attenuated the phenotypes of tumor formation related phenotypes.

To summary, our data, together with others’ publications, suggested a critical role of ID1 in the development and progression of PDAC in both mouse models and human PDAC. ID1 expression marked the heterogeneity of human PDAC cancer cells and the relative expression levels were correlated with the malignance of PDAC cancer cell clusters. Targeting ID1 and the upstream regulator remarkably rescued the PDAC cell lines from tumor formation related phenotypes in vitro. These data revealed the potential of ID1 as a biomarker for PDAC diagnosis and prognosis and emphasized again the therapeutic values of ID1 in the prediction and treatment of PDAC in clinical precision medicine.

## Supplementary Information

Supplementary Figures.

## Data Availability

Publicly available datasets were analyzed in the present study. The data can be found on the Gene Expression Omnibus database (GSE125588, GSE129455, GSE85241, GSE141017, GSE16515, GSE28735, GSE32676 and GSE15471) and Sequence Read Archive (PRJNA516878, PRJNA531464, PRJNA337935 and PRJNA604712). All the data used and/or generated in the current study are available from the corresponding author upon reasonable request.

## Abbreviations

scRNA-seq: Single cell RNA-sequencing
PDAC: Pancreatic ductal adenocarcinoma
ID1: Inhibitor of DNA binding 1
EIF2A: Eukaryotic translation initiation factor 2A
EDU: 5-Ethynyl-2ʹ-deoxyuridine
KIC: Kras^LSL−G12D/+^Ink4a^fl/fl^Ptf1a^Cre/+^
KPfC: Kras^LSL−G12D/+^Trp53^fl/fl^Pdx1^Cre/+^
KPC: Kras^LSL−G12D/+^Trp53^LSL−R172H/+^Ptf1a^Cre/+^
KPC2: Kras^LSL−G12D/+^Trp53^LSL−R172H/+^Pdx1^Cre/+^
IPA: Ingenuity Pathway Analysis
EMT: Epithelial-mesenchymal transition
