# Supplementary material for: ID1 marks the tumorigenesis of pancreatic ductal adenocarcinoma in mouse and human
Source: Sci Rep. 2022 Aug 8;12:13555. doi: 10.1038/s41598-022-17827-3 (PMC9359991; doi:10.1038/s41598-022-17827-3)
Supplement: Supplementary file 1 — Supplementary Figures. [file 41598_2022_17827_MOESM1_ESM.pdf]

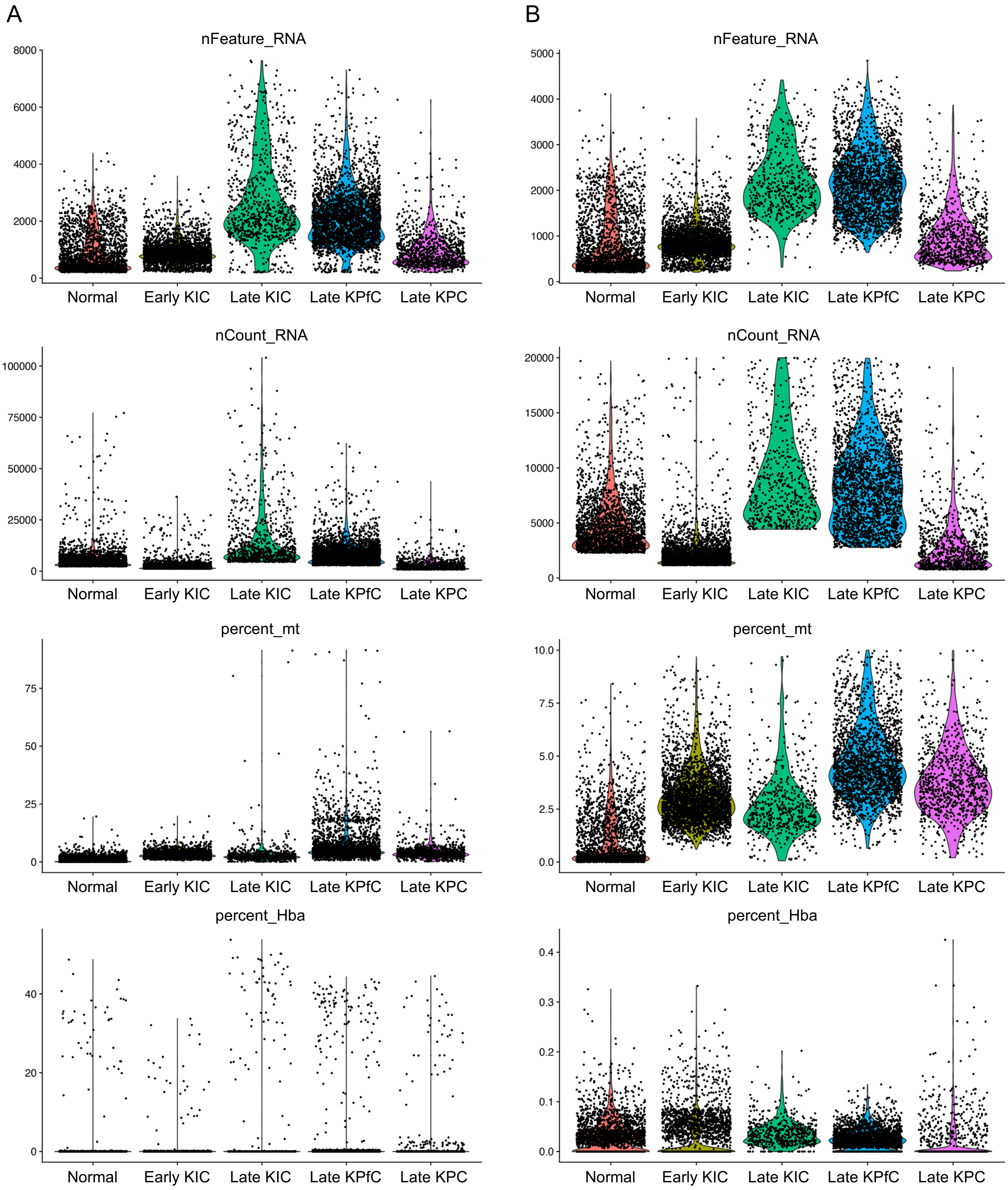

Figure S1. Violin plot visualization of the cells of each mouse model in scRNA-seq data from GSE125588 before (**A**) and after (**B**) quality control.

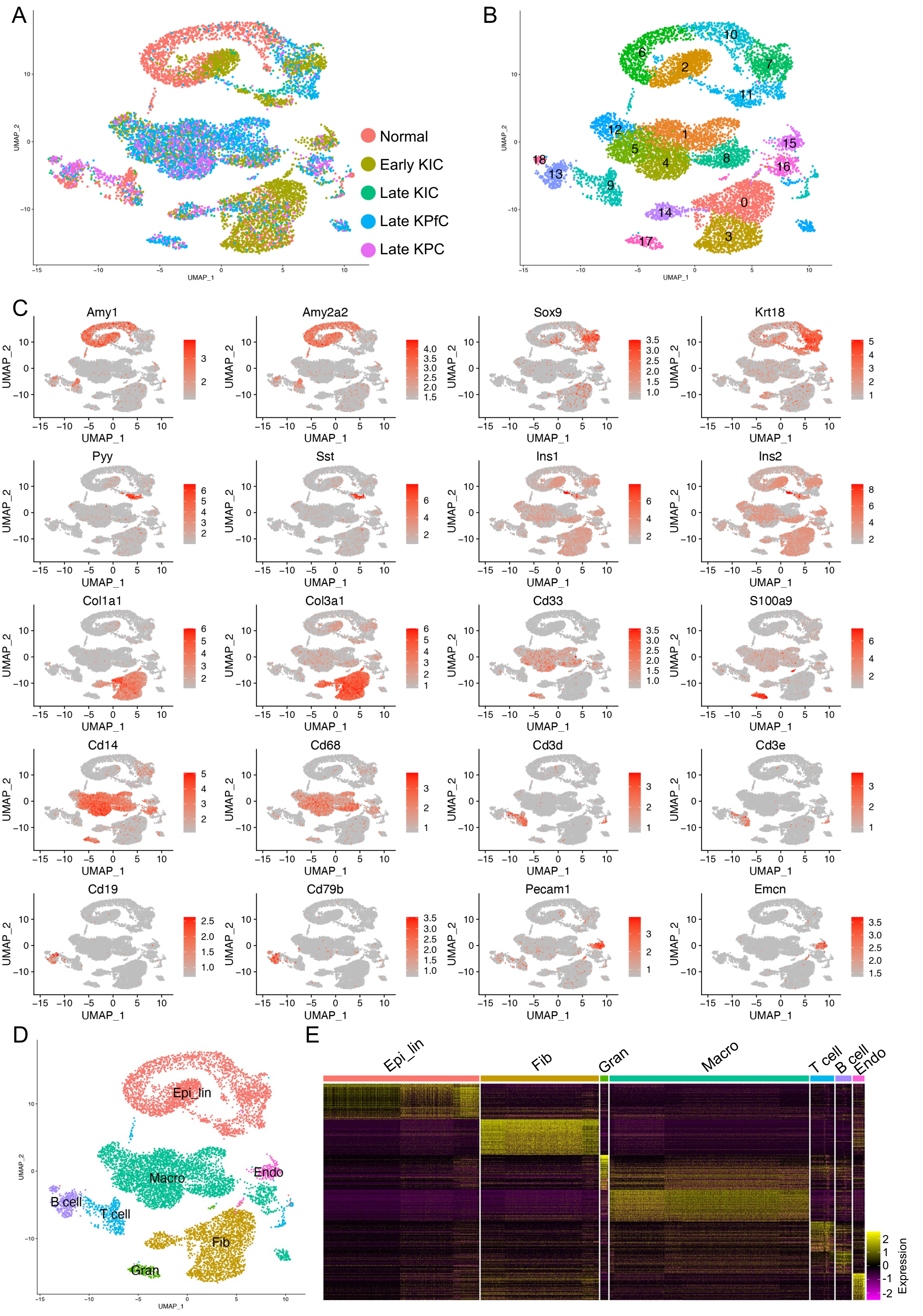

Figure S2. Cell type identification of the integrated data of the cell from normal pancreas, early KIC, late KIC, late KPfC and late KPC mouse models. (**A**) Cell distribution cells from each mouse model. (**B**) Clustering of the cells in the integrated data. (**C**) UMAP visualization of the expression of canonical cell type markers. (**D**) Cell type definition of the integrated data based on the canonical cell type marker expression in (**C**). (**E**) Heatmap of top 100 genes of each defined cell type in the integrated data.

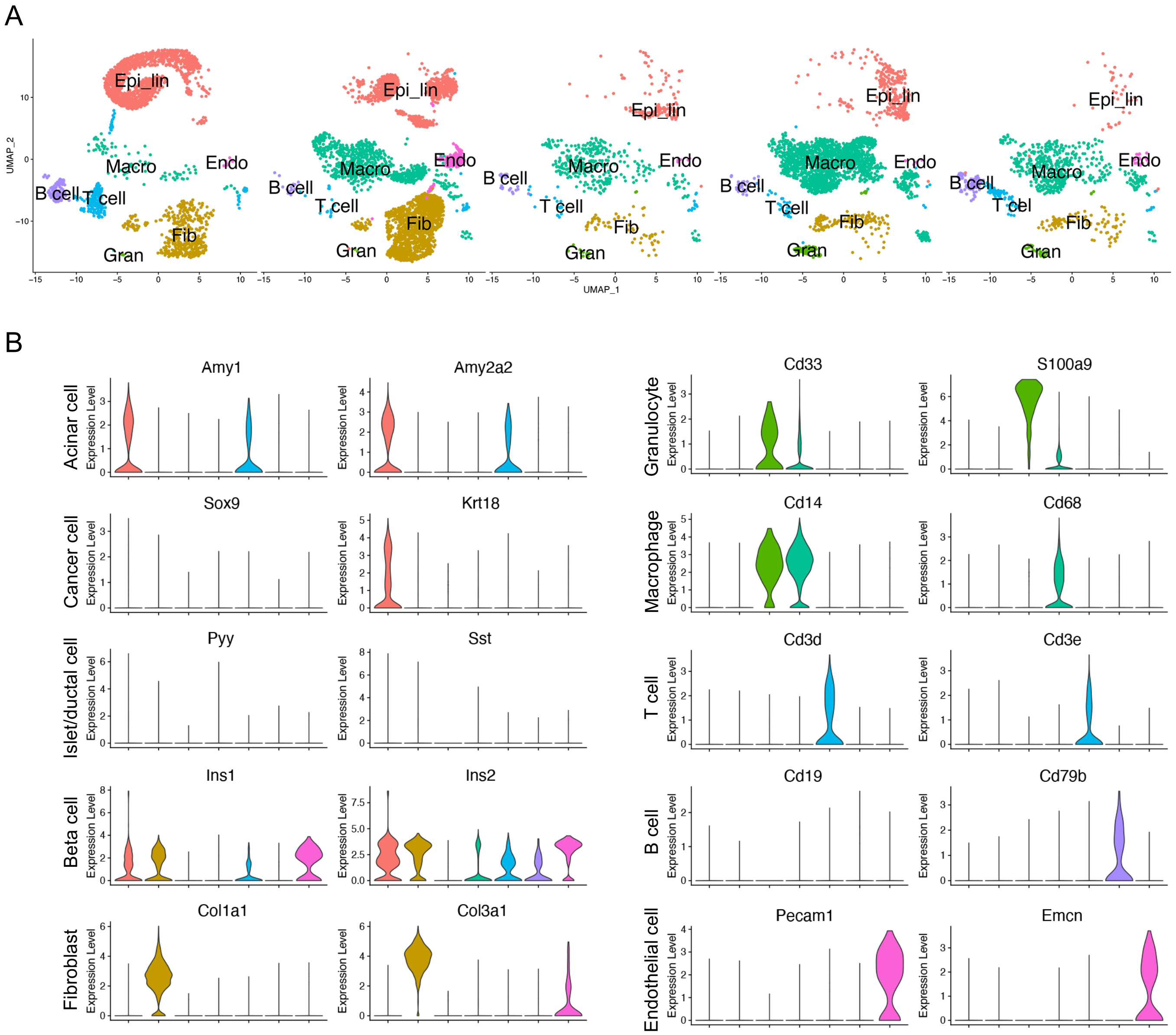

Figure S3. Contribution of each mouse model to the defined cell types in the integrated data. (**A**) Cell distribution of each cell type in each mouse model visualized by split UMAP. (**B**) Validation of the cell type definition by canonical cell type marker expression visualized by violin plot split by models.

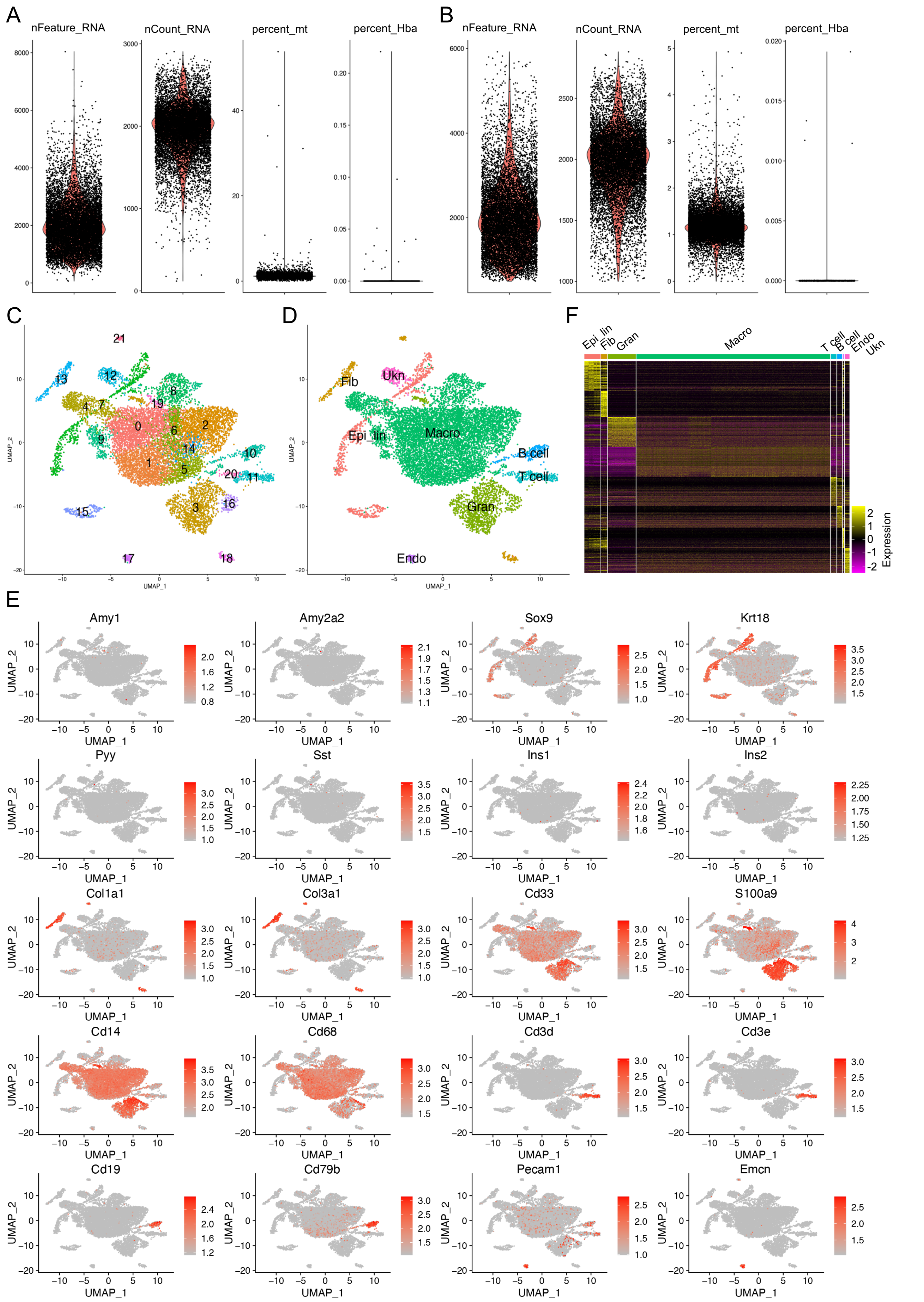

Figure S4. Reuse of the scRNA-seq data on another late mouse KPC (KPC2) model accessed from GSE129455. (**A**-**B**) Violin plot visualization of the cell qualities before (**A**) and after (**B**) quality control. (**C**-**D**) Cell clustering (**C**) and the cell type definition (**D**) of the cells in late KPC2 mouse model. (**E**) The expression of the canonical cell type markers visualized by UMAPs. (**F**) Heatmap of top 100 genes of each defined cell type in late KPC2 mouse model.

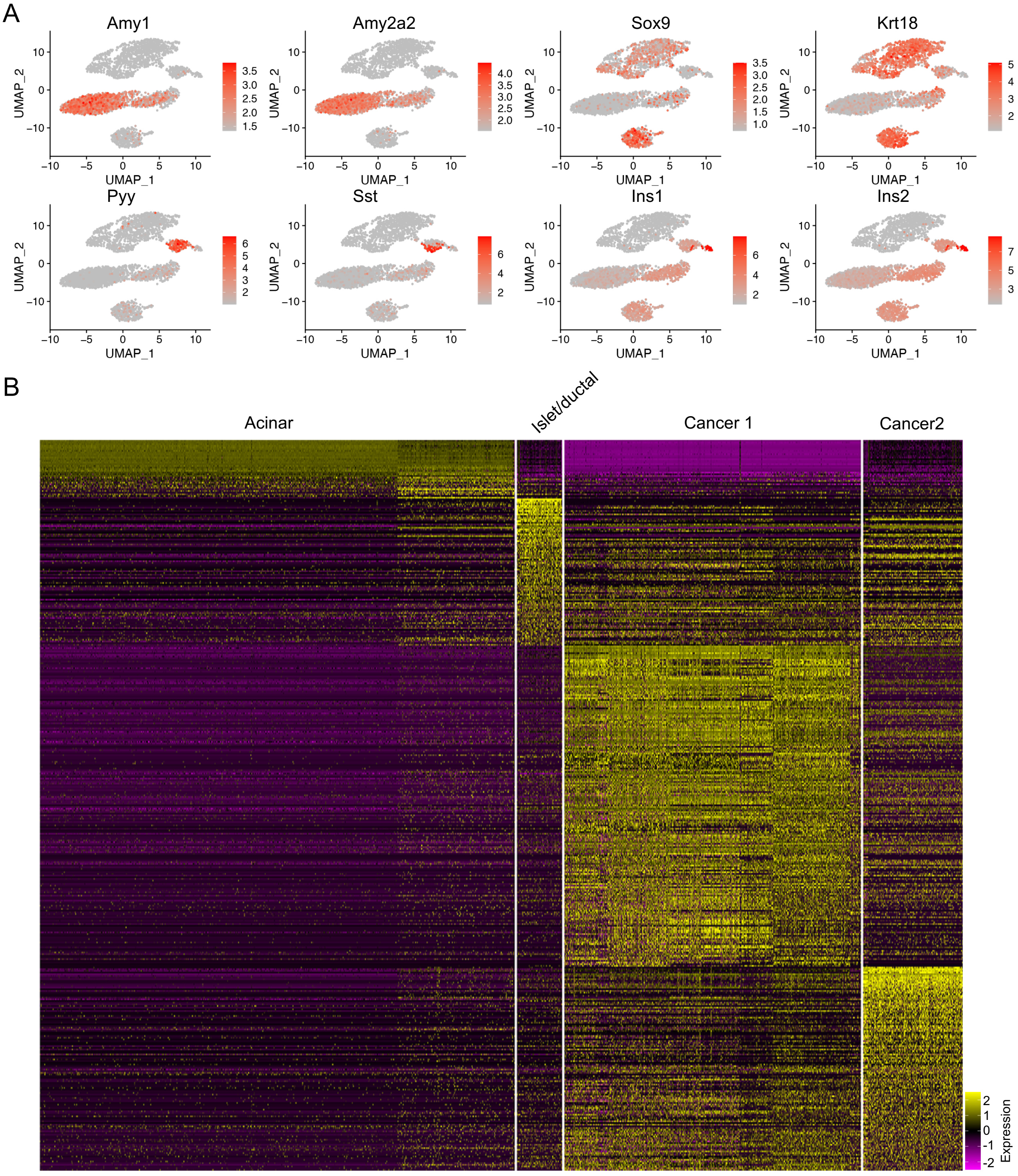

Figure S5. Cell type identification of the epithelial lineage cells in the integrated data of normal pancreas, early KIC, late KIC, late KPfC, late KPC and late KPC2 mouse models. (**A**) Expression of canonical epithelial lineage markers and cancer related markers in the integrated data. (**B**) Heatmap of top 200 genes of each defined epithelial cell lineage and cancer cell cluster in the integrated data.

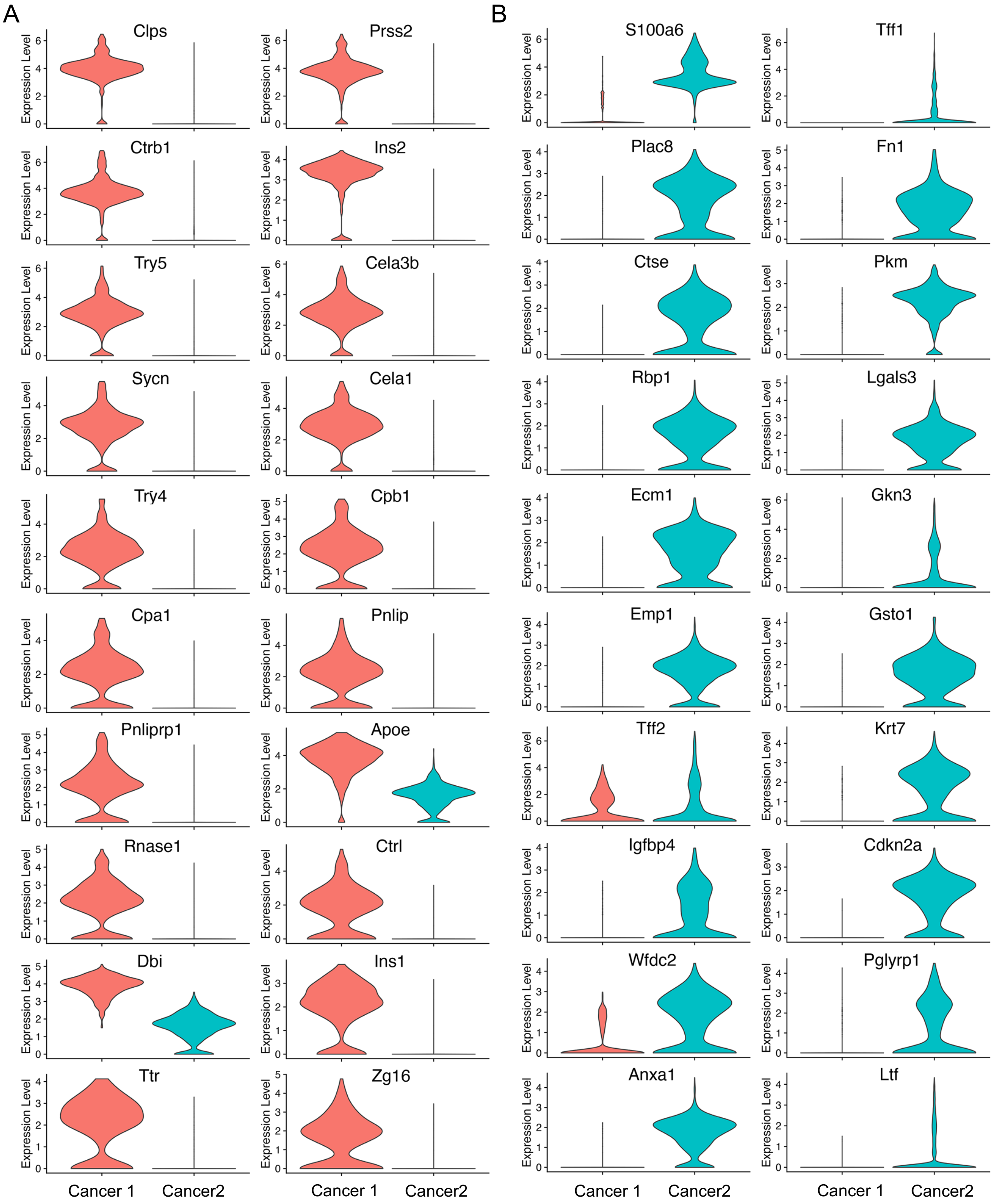

Figure S6. Top 20 genes of each cancer subclusters were visualized by violin plots: Cancer cluster 1 (**A**) and Cancer cluster 2 (**B**).

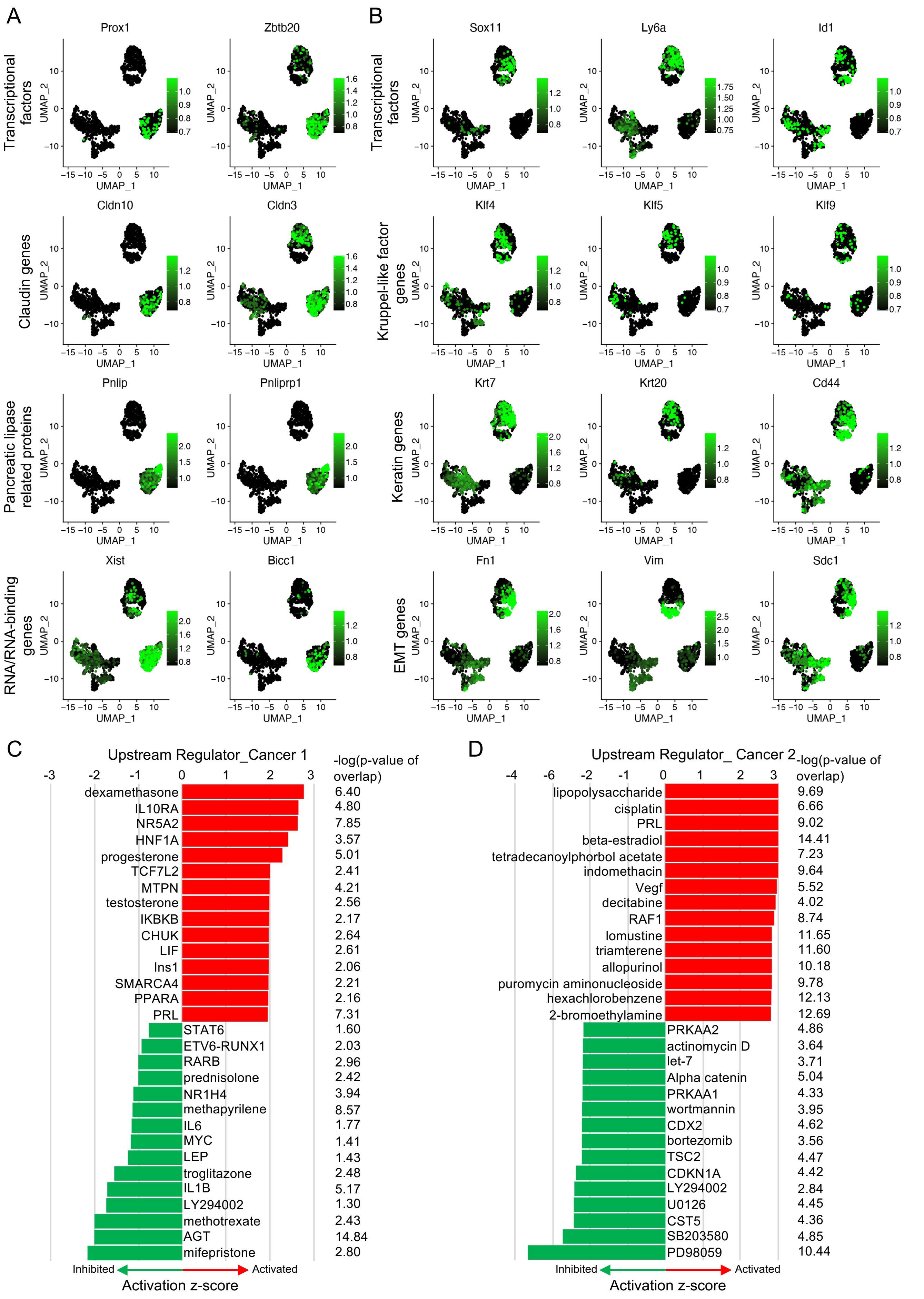

Figure S7. The expression of genes closely associated with the progression of mouse PDAC. (**A-B**) UMAP visualization of the expression of genes specific in Cancer cluster 1 (**A**) and Cancer cluster 2 (**B**) in the data clustered in Slingshot packages. (**C-D**) Upstream Regulators of Cancer cluster 1 (**C**) and Cancer cluster 2 (**D**) determined by IPA analysis.

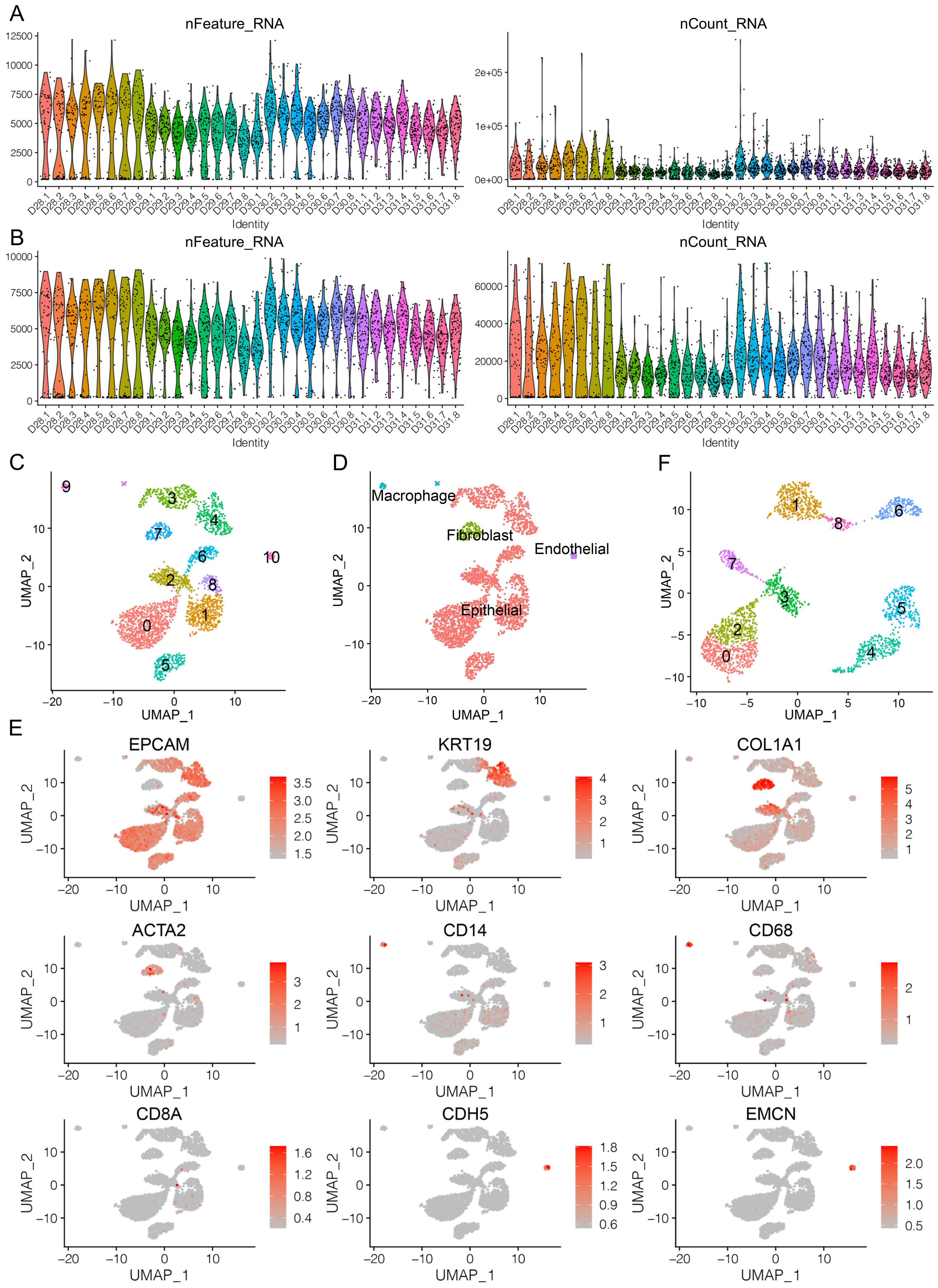

Figure S8. Quality control and cell type identification of scRNA-seq data on normal human pancreases tissues from GSE85241. (**A-B**) Violin plot visualization of the cell qualities before (**A**) and after (**B**) quality control. (**C-D**) Cell clustering (**C**) and the cell type definition (**D**) of the cells of normal human pancreases tissues. (**E**) The expression of the canonical cell type markers visualized by UMAPs. (**F**) Extraction of epithelial cells from the scRNA-seq data on normal human pancreases tissues.

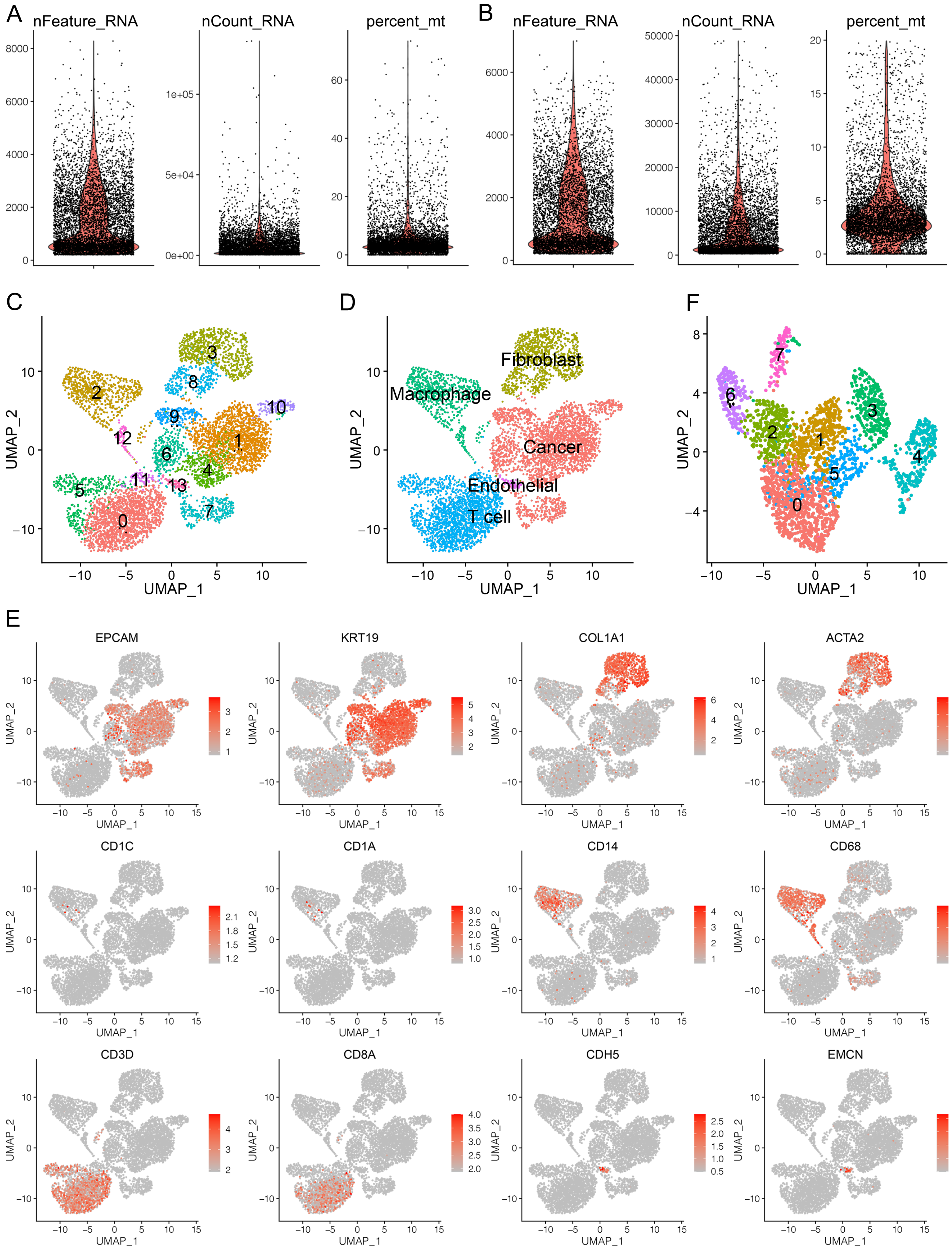

Figure S9. Quality control and cell type identification of scRNA-seq data on human PDAC from GSE141017. (**A**-**B**) Violin plot visualization of the cell qualities before (**A**) and after (**B**) quality control. (**C**-**D**) Cell clustering (**C**) and the cell type definition (**D**) of the cells of human PDAC tissues. (**E**) The expression of the canonical cell type markers visualized by UMAPs. (**F**) Extraction of epithelial cells from the scRNA-seq data on human PDAC tissues.

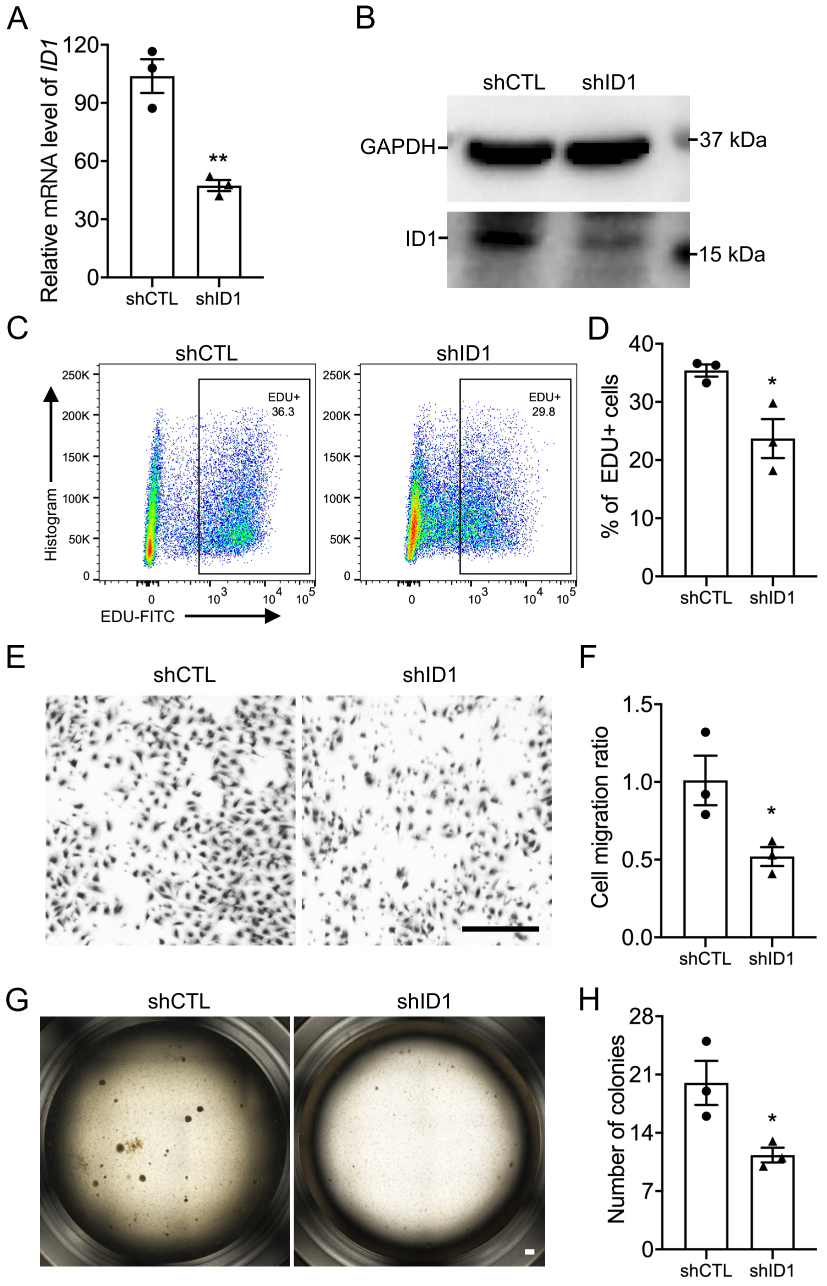

Figure S10. ID1 deficiency attenuated tumor formation related phenotypes in PANC-1 cells. (**A**) qRT-PCR and (**B**) Western blotting for ID1 in PANC-1 cells after knockdown assays (n = 3). (**C-D**) Flow cytometry analysis of EDU labeled cell proliferation assay in PANC-1 cells after knockdown assays (n = 3). (**E-F**) Cell migration and the migration ratio of PANC-1 cells after ID1 knockdown (n = 3). (**G-H**) Soft agar assays and colony number quantification of PANC-1 cells after ID1 knockdown (n = 3). Three independent repeats were performed for each experiment. Scale bar, 50 μm. *, $p < 0.05$; **, $p < 0.01$.

Full-length blots Figure 8B

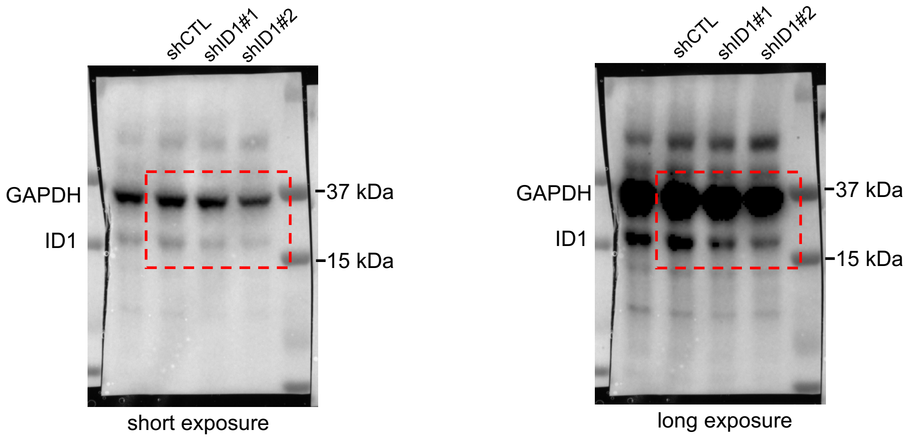

Full-length blots Figure 9E and G

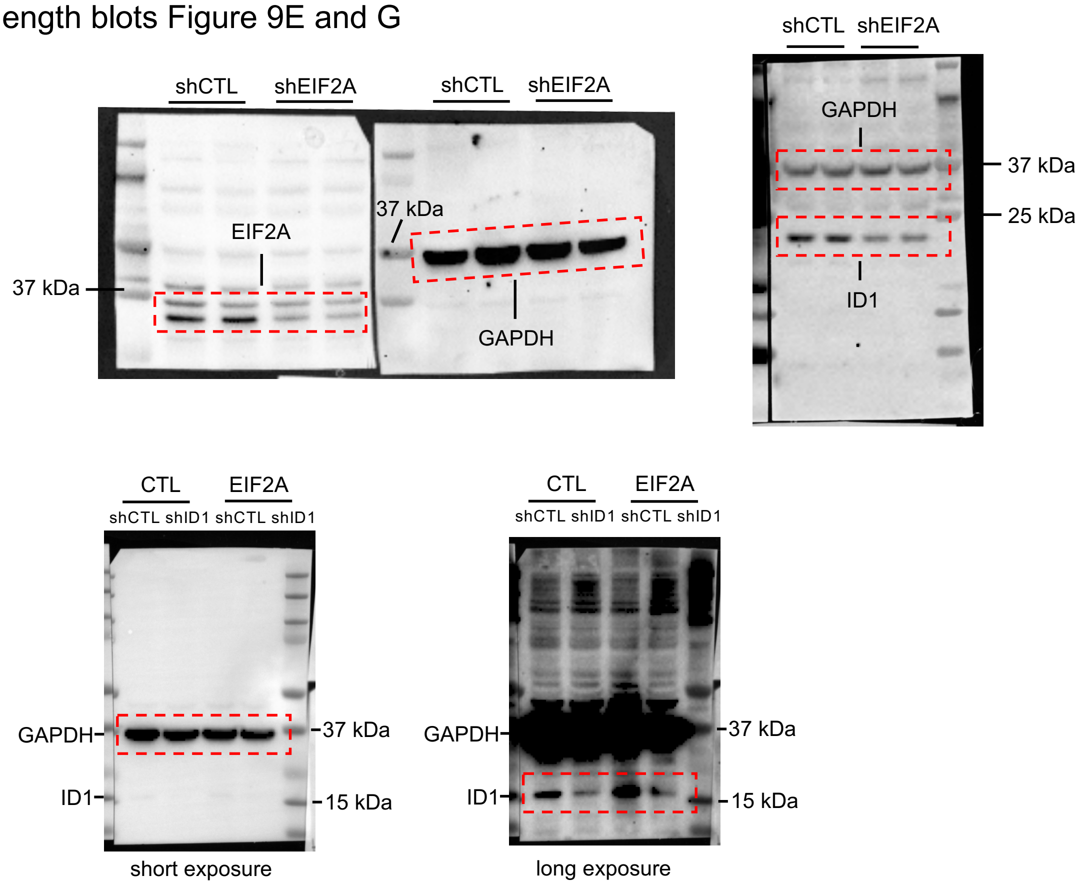

Full-length blots Figure S10B

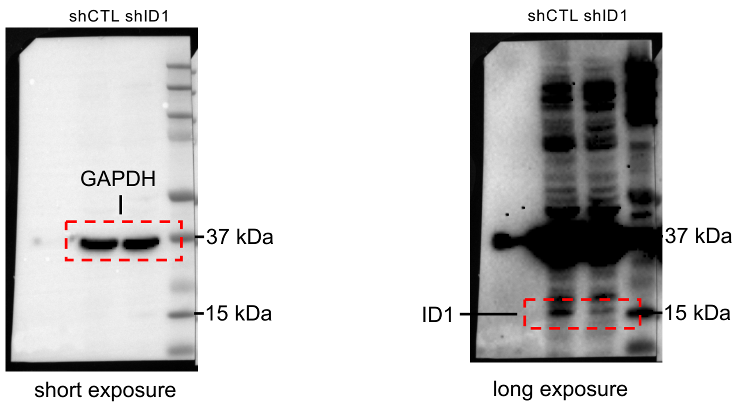

Figure S11. Full-length uncropped gels for main Figure 7F and 8D.
